# Bioinformatics-based discovery of biomarkers and immunoinflammatory targets in children with cerebral palsy: An observational study

**DOI:** 10.1097/MD.0000000000037828

**Published:** 2024-04-19

**Authors:** Bo Chen, Ling Wang, Dongke Xie, Yuanhui Wang

**Affiliations:** a Department of Rehabilitation, The Affiliated Hospital of Southwest Medical University, Southwest Medical University, Luzhou, China; b Department of Rehabilitation Science, Hong Kong Polytechnic University, Hong Kong, China; c Department of Operating Room, The Affiliated Hospital of Southwest Medical University, Southwest Medical University, Luzhou, China; d Pediatric Surgery, The Affiliated Hospital of Southwest Medical University, Southwest Medical University, Luzhou, China; e Sichuan Clinical Research Center for Birth Defects, The Affiliated Hospital of Southwest Medical University, Southwest Medical University, Luzhou, China.

**Keywords:** Bioinformatics analysis, biomarkers, CP, hypoxia, inflammation

## Abstract

Cerebral palsy (CP) is the most common disabling disease in children, and motor dysfunction is the core symptom of CP. Although relevant risk factors have been found to be closely associated with CP: congenital malformations, multiple gestation, prematurity, intrauterine inflammation and infection, birth asphyxia, thrombophilia, and perinatal stroke. Its important pathophysiological mechanism is amniotic fluid infection and intraamniotic inflammation leading to fetal developing brain damage, which may last for many years. However, the molecular mechanism of CP is still not well explained. This study aimed to use bioinformatics to identify key biomarker-related signaling pathways in CP. The expression profile of children with CP was selected from the Gene Expression Comprehensive Database, and the CP disease gene data set was obtained from GeneCards. A protein–protein interaction network was established and functional enrichment analysis was performed using Gene Ontology and Kyoto Encyclopedia of Genes and Genomes databases. A total of 144 differential key intersection genes and 10 hub genes were identified through molecular biology. Gene Ontology functional enrichment analysis results show that differentially expressed genes are mainly concentrated in biological processes, such as immune response and neurogenesis. The cellular components involved mainly include axons, postsynaptic membranes, etc, and their molecular functions mainly involve proteoglycan binding, collagen binding, etc. Kyoto Encyclopedia of Genes and Genomes analysis shows that the intersection genes are mainly in signaling pathways related to the immune system, inflammatory response, and nervous system, such as Th17 cell differentiation, Toll-like receptor signaling pathway, tumor necrosis factor signaling pathway, NF-κB signaling pathway, axon guidance, PI3K-Akt signaling pathway, HIF-1 signaling pathway, gap junction, etc. Jak-STAT signaling pathway, mTOR signaling pathway, and related hub genes regulate immune cells and inflammatory factors and play an important role in the development and progression of CP.

## 1. Introduction

Cerebral palsy (CP) is the most common disabling disease in children. It is a nonprogressive damage to the developing brain that causes permanent developmental disorders of movement and posture, which in turn leads to limitations in the patient’s activities. Among them, motor dysfunction is the core symptoms of CP.^[1]^ Random sampling surveys in some areas of my country show that the prevalence of CP among children aged 0 to 6 years old is 2.37‰. CP puts great pressure on the medical security system and families. At the same time, the quality of life of CP patients is also extremely seriously affected.^[2]^ Its clinical features include impairments in movement and posture, motor deficits, lack of coordination, and spasms involving upper motor neurons. Movement disorders in CP are often accompanied by other related disorders, such as epilepsy, intellectual disability, and sensory impairment.^[3]^ Some studies have reported that acute intrapartum hypoxia-ischemia is the main cause of CP.^[4]^ Risk factors associated with CP include congenital malformations, multiple gestations, prematurity, intrauterine inflammation and infection, birth asphyxia, thrombophilia, and perinatal stroke. An important pathophysiological mechanism observed is that infection of the amniotic fluid and intraamniotic inflammation leads to damage to the developing fetal brain, leading to CP, damage that may persist for many years.^[5]^ Children with CP generally have a poor quality of life. To date, the pathogenesis of CP in children has not been fully understood. It is generally believed that CP is related to the combined effects of multiple perinatal factors, and hypoxia may be the key link.^[6,7]^

Studies have found that IL-6, IL-1β, IL-8, and TNF-α are the protein biomarkers that best predict neurodevelopmental damage. IL-6 is an independent predictor of.^[8,9]^ In addition, plasma TNF-α levels in CP patients were significantly higher than those in normal controls, and TNF-α levels were significantly correlated with disease severity. IL-1β plays a key role in the neuroinflammatory mechanism of ischemic–hypoxic encephalopathy.^[10]^ Studies have confirmed that carrying a single nucleotide polymorphism at position 511 of the IL-1β gene promoter and the amplification of the CCTTT microsatellite in the NOS2A promoter may make children more susceptible to ischemic–hypoxic encephalopathy. CP occurs. It can be seen that hypoxia and its resulting inflammatory mediators play an important role in the occurrence of CP.^[11]^

There is currently no treatment that can cure CP. Early diagnosis and intervention for CP, and exploring differentially expressed genes and treatment targets related to CP are of great significance for reducing the incidence of CP, improving patient prognosis, and reducing disability rates. In recent years, with the rapid development of gene chips and high-throughput sequencing technology, researchers can quickly achieve comprehensive and detailed analysis of transcriptomes and genomes, which has effectively promoted the development and progress of life sciences.^[12,13]^ With the increasing number of related studies on CP gene expression profiles and the establishment of various public databases year by year, bioinformatics methods can be used to conduct deeper research on CP, thereby expanding the understanding of the pathological mechanisms of CP.^[14]^ This study collected genetic data from children with CP to obtain differentially expressed genes of CP. Based on this, bioinformatics analysis was performed and the core driver genes of CP were screened out to provide direction for further research.

## 2. Materials and methods

### 2.1. Microarray data source and differential gene analysis

Data set GSE183021 was downloaded from the Gene Expression Omnibus (GEO) database. The chip data of GSE183021 were studied on 5 children with CP and 5 healthy twins to eliminate interfering factors (average age: 3.3 ± 1.5, average birth weight: 2.9 ± 0.4 kg).^[14]^ GPL29703 is a sequencing data platform for data, all of which use the human body as the source.^[14]^ Use the “limma” tool of Sangerbox 3.0 to perform differential gene analysis to clarify the differential genes between CP samples and control samples to study the impact of differences in related gene expression levels in CP patients.^[15]^ The threshold for differential genes was set to a 1.5-fold difference and *P* value < .05. Positive values of logFC indicate upregulation of differentially expressed genes (DEGs). Similarly, a fold difference of 1.5-fold and *P* value < .05, and a negative numerical value of logFC indicates downregulation of DEGs. Volcano plots and heat maps were used to display the results of DEGs. Among them, the difference heat map mainly shows the top 30 genes with large differences.

### 2.2. Weighted gene co-expression network analysis (WGCNA)

WGCNA package of Sangerbox 3.0 was used to construct the gene co-expression network of GSE183021. According to the clustering tree, the top 50% of genes with the smallest MAD were eliminated. Calculate the correlation coefficient between each gene pair and construct a similarity matrix. To ensure the construction of a scale-free network, a suitable soft threshold is chosen to convert the similarity matrix into an adjacency matrix. Subsequently, a topological overlap matrix (TOM) was created to measure the average network connectivity of each gene. Based on the relevant parameters of the block-dimensional module function, a dynamic tree-cutting method was used to group genes with similar expression profiles into different modules. Each module is depicted with a different color, where genes in gray modules represent genes that cannot be assigned to any module. The gene expression profile of each module consists of the first principal component called module eigengene (ME). MEs are used to evaluate the association between modules and phenotypes. The module with the highest absolute value of the correlation coefficient is determined as the key module for further analysis. Module membership (MM) is the correlation coefficient between the expression value of a gene and the ME of a module, indicating the correlation between the gene and the module. Gene significance (GS) is the correlation coefficient between the expression value of a gene and a phenotype, representing the correlation between the gene and the phenotype. Set the MM threshold to 0.8, the GS threshold to 0.1, and the weight threshold to 0.1 to extract hub genes.^[16]^

### 2.3. Acquisition of CP-related disease data sets

To further study the targets and functions of diseases in children with CP, using “CP” as the keyword, we published (https://www.genecards.org/), search the disease database to obtain the target proteins of the disease.^[17,18]^ The search results of these databases were merged and deduplicated to obtain the CP disease gene set.

### 2.4. CP biomarker identification

To accurately identify the key biomarkers that play a role in the process of CP, we intersected the differential gene set and module gene set of the CP disease gene data set and the GEO integrated data set to obtain the intersection gene set. This intersection gene set is an important gene in the process of CP. Protein–protein interactions underlie most biological processes in living cells and are critical to understanding cellular physiology in normal and disease states. In this study, the string database (http://string-db.org/) was used to perform PPI network analysis on the obtained intersection gene set, and the species was restricted to “*Homo sapiens*” with a confidence value > 0.4. The PPI network was constructed by Cytoscape software (version 3.9.1).^[19]^ In addition, the key gene set was obtained.^[20]^ To further pinpoint the genes involved in the process of CP, the CytoHubba algorithm was used to screen key gene sets, and finally, the hub genes with significant effects were obtained. Put the hub genes into the GEO integrated data set to find and extract the differential gene expression between CP samples and normal samples, and use box plots to display the results of differential gene expression.

### 2.5. Enrichment analysis

Import the hub gene set into the Sangerbox 3.0 biological cloud platform, select enrichment analysis in the tool center, and limit the species to “*H. sapiens*.” Enter the Gene Symbol of the hub gene set in the common parameters and submit. Finally, we obtained Gene Ontology (GO) enrichment analysis and Kyoto Encyclopedia of Genes and Genomes (KEGG) database pathway analysis of hub genes and displayed the results in different charts.^[21,22]^

## 3. Result

### 3.1. Differential gene analysis results based on different groups

**Figure 1.**
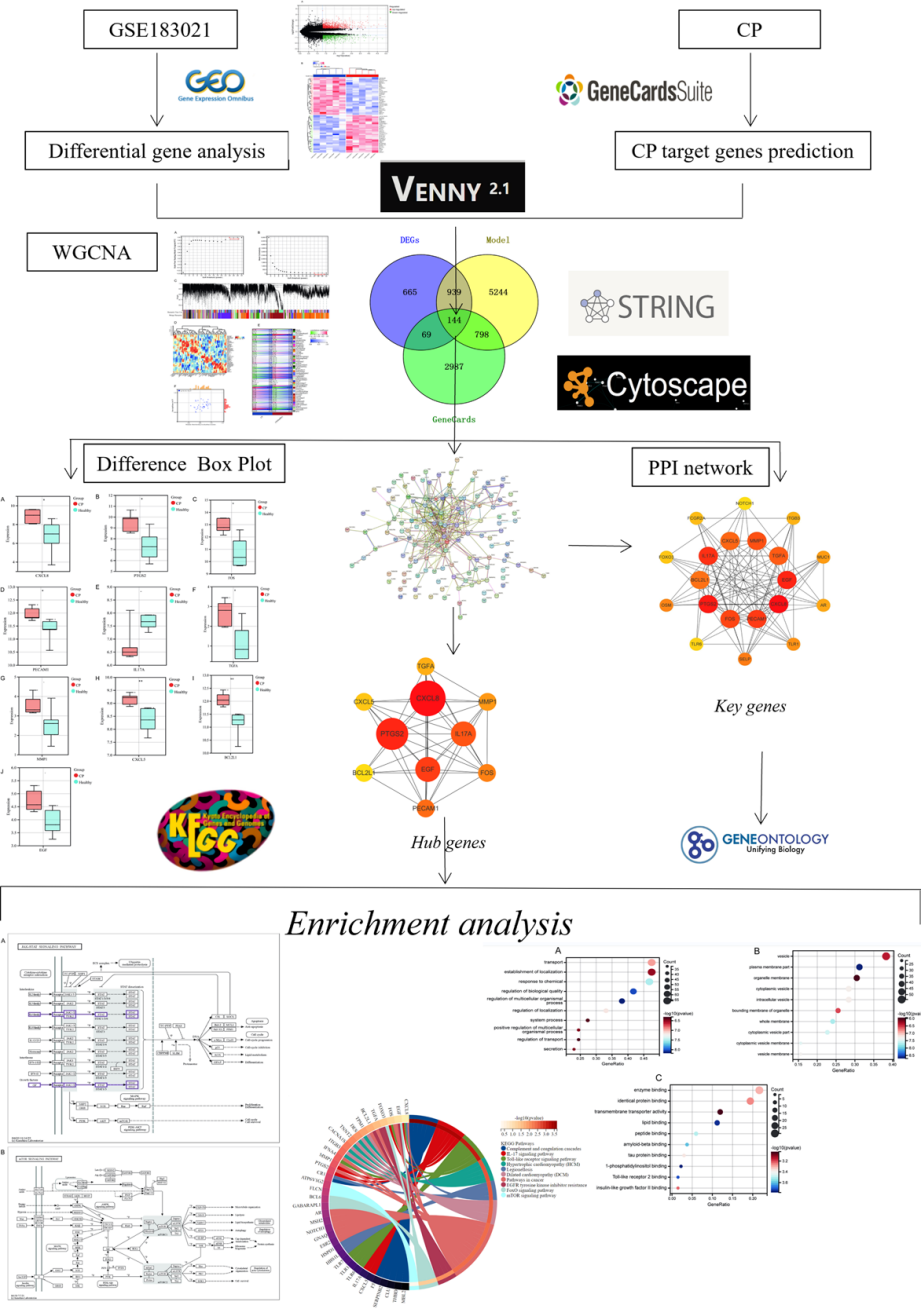
Process structure relationship of the article.

**Figure 2.**
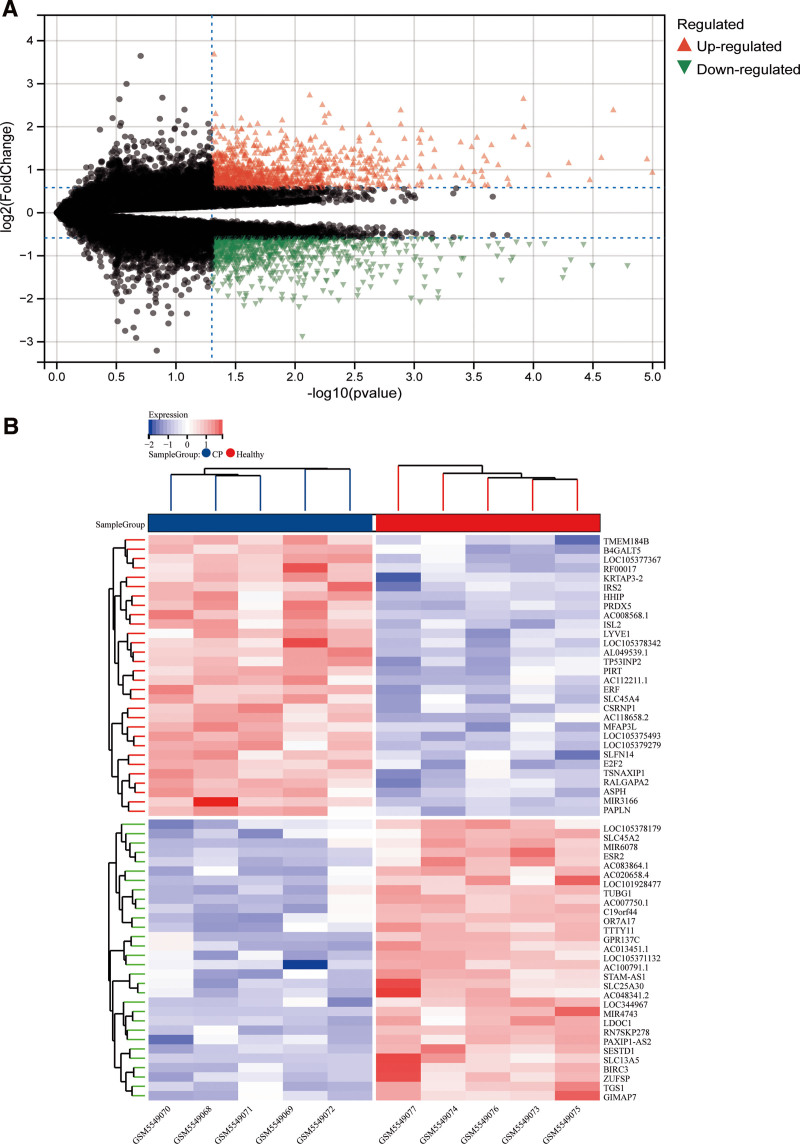
Limma analysis is a differential expression screening method based on generalized linear models. (A) The difference volcano plot shows the differential gene expression as a whole. The red triangle represents the upregulated genes, the green triangle represents the downregulated genes, and the gray triangle represents the nonsignificantly different genes. (B) The difference heat map clearly shows the significant differences between the groups. Genes, red indicates upregulation, blue indicates downregulation.

The bioinformatics analysis process of this study is shown in Figure 1. After downloading and processing from the GEO data set, GSE183021 contains 5 CP samples and 5 normal control samples. The study of differences between CP samples and control samples showed that there were 1817 DEGs among 29,665 genes, of which 966 genes were upregulated and 851 genes were downregulated (Table S1, Supplemental Digital Content, http://links.lww.com/MD/M175 and Table S2, Supplemental Digital Content, http://links.lww.com/MD/M176, Fig. 2A and B).

### 3.2. WGCNA results

**Figure 3.**
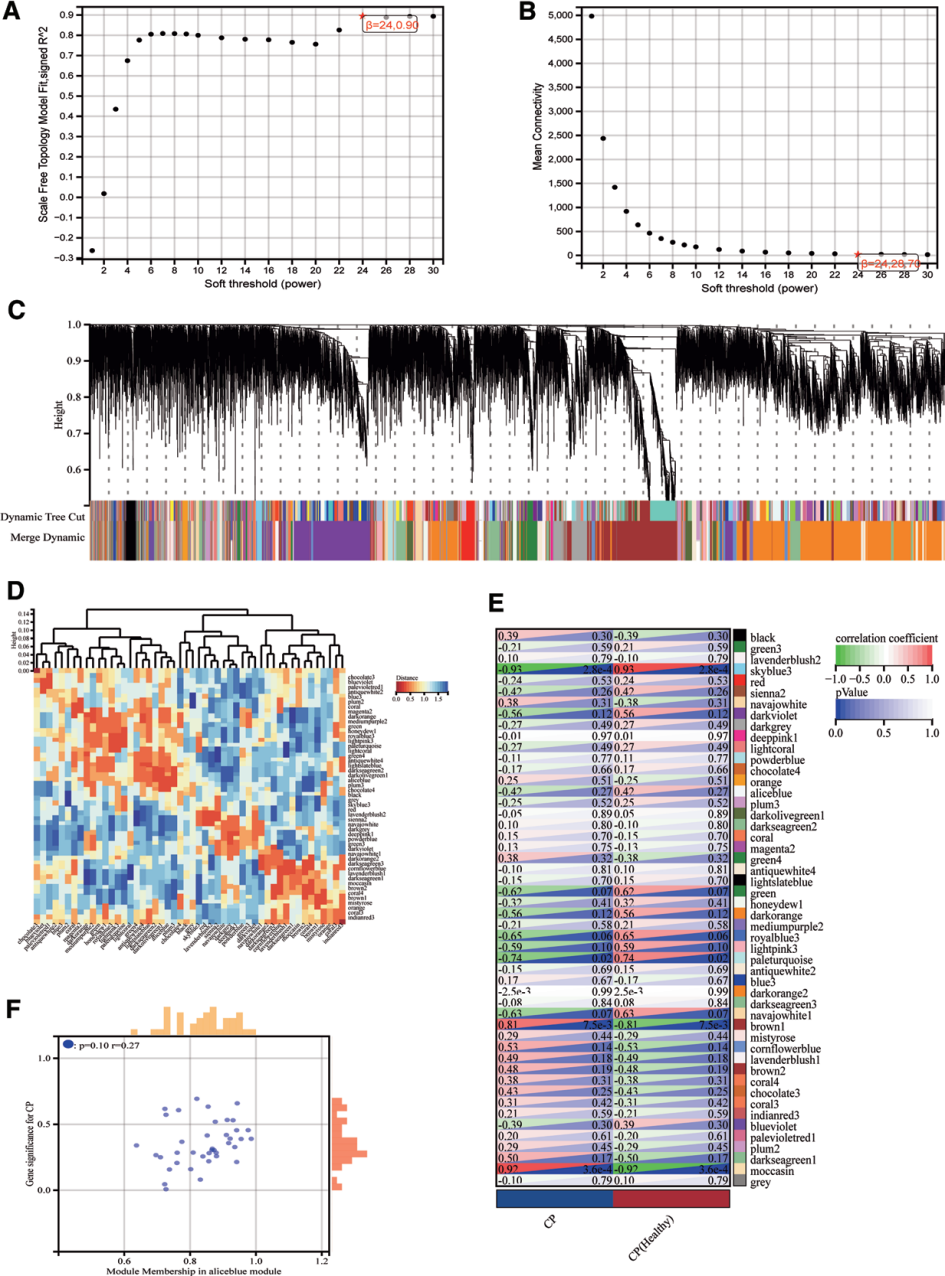
Results of WGCNA. (A) Corresponding scale-free topological model fitted fitting index at different soft threshold powers, (B) corresponding average connectivity values at different soft threshold powers, (C) clusters of genes Dendrogram, (D) cluster analysis of module feature vectors of each module, (E) correlation analysis of modules with clinical characteristics, (F) correlation analysis between MM and GS. GS = gene significance, MM = module membership, WGCNA = weighed gene co-expression network analysis.

After removing abnormal samples and screening genes, the expression profiles of 29,665 genes were extracted from GSE18302 1 and used to construct a weighted gene co-expression network. When the soft threshold power was set to 24, the scale independence reached 0.90 and the average connection value was 28.70 (Fig. 3A, B). When the cutting height was set to 0.25 and the minimum module size was set to 30, 50 different co-expression modules were obtained through dynamic tree cutting (Fig. 3C). Module feature vector cluster analysis of each module shows that the plum3 and darkseagreen2 modules have the largest distance (Fig. 3D). Then, the correlation analysis between each module and clinical characteristics were performed. The plum3 module was positively correlated with CP (correlation coefficient = 0.25, *P* = .52), and the darkseagreen2 module was negatively correlated with CP (correlation coefficient = −0.10, *P* = .80; Fig. 3E). In addition, correlation analysis between MM and GS showed that these genes were highly correlated with both modules and phenotypes (*R* = 0.27, *P* = .10 Fig. 3F). Finally, after all module genes were extracted, we obtained 7125 module genes (Table S3, Supplemental Digital Content, http://links.lww.com/MD/M177).

### 3.3. CP biomarker identification

**Figure 4.**
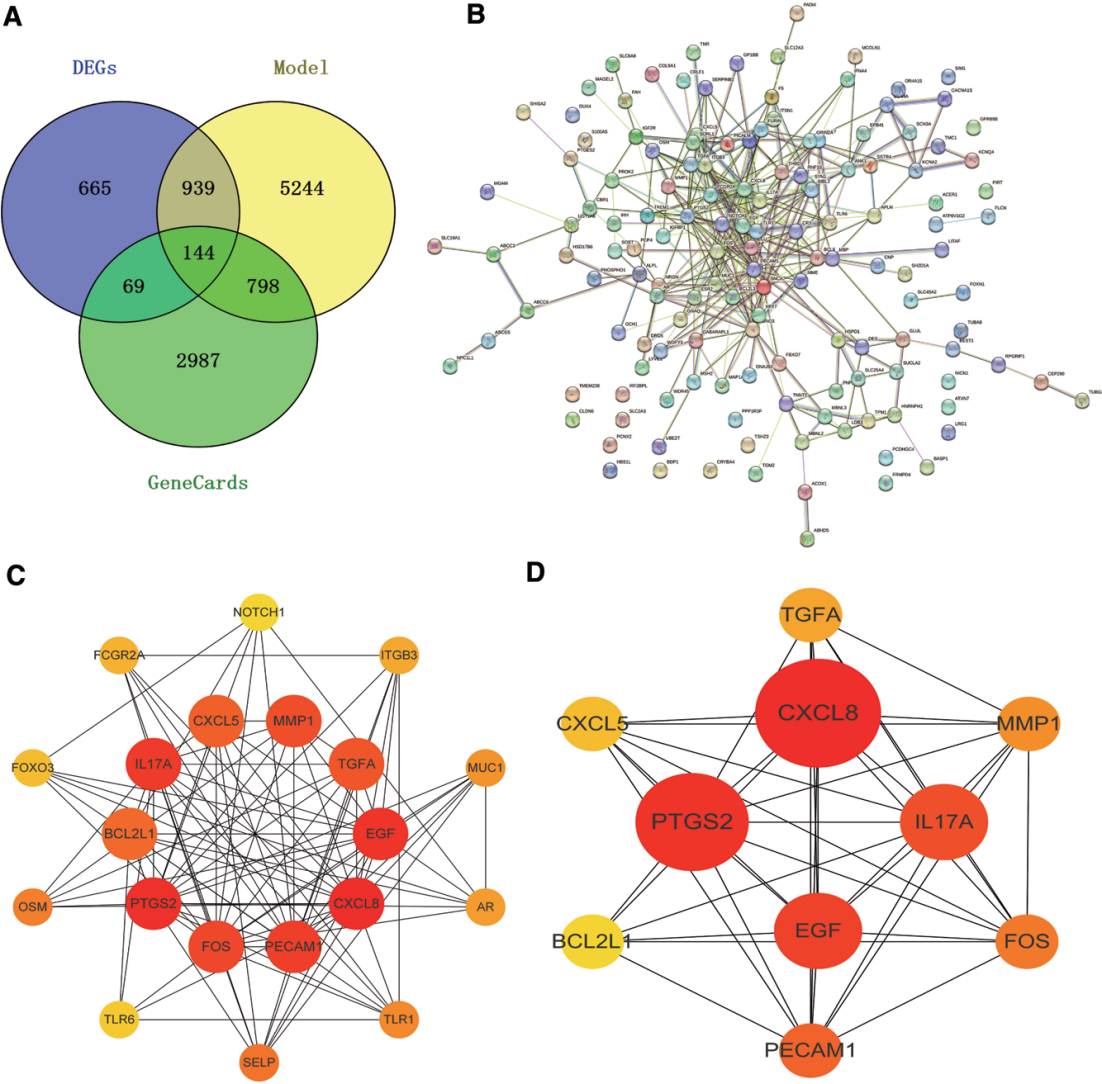
(A) There are 3998 targets for CP disease, which intersect with 7125 module genes and 1817 differential genes, and finally 144 important intersection gene sets are obtained; (B) 20 key gene sets obtained through PPI screening; (C) 10 hub gene sets obtained through PPI screening. CP = cerebral palsy, PPI = protein–protein interaction.

**Figure 5.**
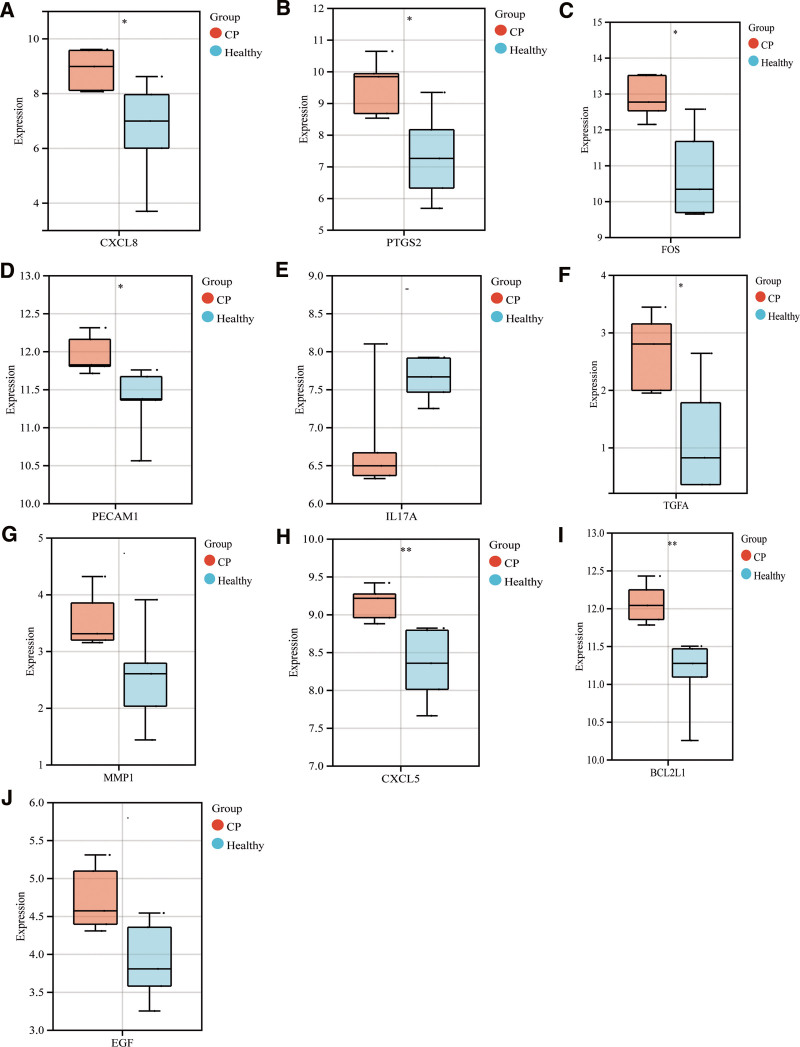
The differential gene boxplot of hub genes shows the statistics of differential expression. (A) CXCL8 (*P < .05), (B) PTGS2 (*P < .05), (C) FOS (*P < .05), (D) PECAM1 (*P < .05), (E) IL17A (NS), (F) TGFA (*P < .05), (G) MMP1 (*P < .05), (H) CXCL5 (**P < .01), (I) BCL2L1 (**P < .0), (J) EGF (*P < .05).

in the GeneCards disease database, and 3998 targets of CP were obtained, which intersected with 7125 module genes and 1817 differential genes, and finally obtained 144 important intersection gene sets (Tables S4 and S5, Supplemental Digital Content, http://links.lww.com/MD/M178, http://links.lww.com/MD/M179, Fig. 4A). Through PPI and Cytoscape screening, 20 key gene sets and 10 hub genes were obtained (Fig. 4B–D). They are CXCL8, EGF, PTGS2, FOS, PECAM1, IL17A, TGFA, MMP1, CXCL5, BCL2L1 (Table S6, Supplemental Digital Content, http://links.lww.com/MD/M180). After comparing with the original expression difference data, we analyzed the expression of 10 hub genes. The differential gene box plot shows the statistics of differential expression of hub genes (Fig. 5A–J). The statistically significant hub genes are CXCL8 and PTGS2, FOS, PECAM1, TGFA, CXCL5, and BCL2L1.

### 3.4. Enrichment analysis

**Figure 6.**
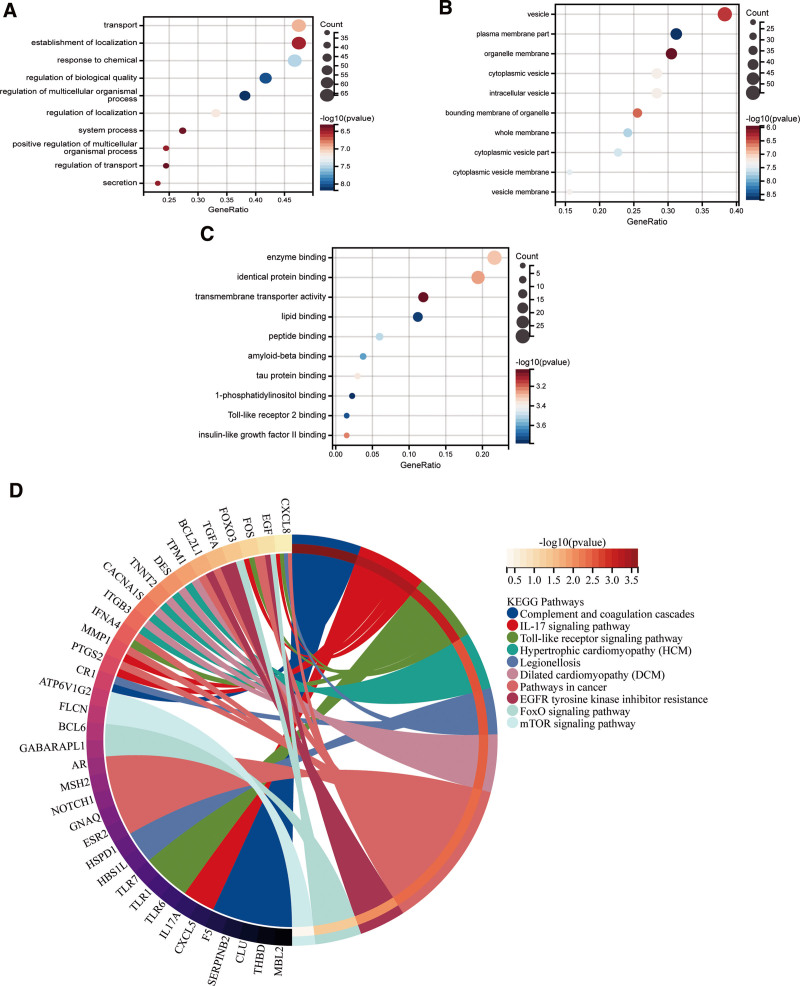
(A) BP enrichment analysis results based on hub genes, (B) CC enrichment analysis results based on hub genes, (C) MF enrichment analysis results based on hub genes, (D) KEGG enrichment circle diagram based on hub genes analyze. BP = biological process, CC = cellular components, KEGG = Kyoto Encyclopedia of Genes and Genomes, MF = molecular_function.

We conducted an enrichment analysis on 144 intersection genes to explore the pathogenesis of CP. GO enrichment analysis showed that hub genes were enriched to a total of 2044 GO entries, including 1000 entries for biological process (BP), 477 entries for cellular component (CC), and 567 entries for molecular_function (MF) (Table S7, Supplemental Digital Content, http://links.lww.com/MD/M181). Among them, the top 10 enrichment results in BP, CC, and MF are displayed in a histogram (Fig. 6A–C). The results show that GO analysis of BP during CP mainly involves transport, establishment of localization, response to chemical, and regulation of biological quality, regulation of multicellular organic process, regulation of localization, system process, positive regulation of multicellular organic process, regulation of transport, secretion. GO analysis CC during CP mainly involves the vesicle, plasma membrane part, organelle membrane, cytoplasmic vesicle, intracellular vesicle, bounding membrane of organelle, whole membrane, cytoplasmic vesicle part, cytoplasmic vesicle membrane, vesicle membrane. GO analysis MF during CP mainly involves enzyme binding, identical protein binding, transmembrane transporter activity, lipid binding, peptide binding, amyloid-beta binding, tau protein binding, 1-phosphatidylinositol binding, Toll-like receptor 2 binding, insulin-like growth factor II binding. KEGG pathway analysis shows that the process of CP involves the expression of 222 signaling pathways (Table S8, Supplemental Digital Content, http://links.lww.com/MD/M182). Screening shows that these pathways mainly involve Complement and coagulation cascades, IL-17 signaling pathway, Toll-like receptor signaling pathway, Hypertrophic cardiomyopathy (HCM), Legionellosis, Dilated cardiomyopathy (DCM), Pathways in cancer, EGFR tyrosine kinase inhibitor resistance, FoxO signaling pathway, mTOR signaling pathway (Fig. 6D). From the perspective of the human body system, CP may be closely related to the immune system, which mainly includes the differentiation of Th 1, Th 2, and Th 17 immune cells.

## 4. Discussion

The specific pathological mechanism of CP is not yet fully understood. It is generally believed that the cause of CP involves multiple joints before, during, and after birth, and is related to hypoxia, premature birth, infection, jaundice, low birth weight, neonatal encephalopathy, and genetics. It is related to other factors, and hypoxia is the main cause of CP.^[23]^ With the help of genomics, molecular genetics, proteomics, and other technical means, people’s understanding and research on CP have continued to deepen. Genetic factors and susceptibility genes have also entered the field of vision of researchers and become a hot area of CP research.^[24,25]^ Genetic factors may change the susceptibility to perinatal inflammation and neurodevelopmental disorders, inducing the occurrence of neurodevelopmental disorders such as CP.^[26]^ In addition, environmental exposures before and after birth can affect the baby’s epigenetic markers, and epigenetic markers early in life can predict neurodevelopmental outcomes years later.^[27]^ Gene-targeted precision therapy has become an important method for the treatment of various malignant tumors. Regulation at the gene level based on epigenetic pathways may also provide promising directions for new treatment strategies for diseases such as CP.^[28,29]^

In this study, we first analyzed the gene expression between children with CP and healthy children through the GEO database and obtained 1817 heterogeneously expressed genes and 7125 module genes. Then, we further analyzed the molecular mechanism of CP by combining these data with the Gene Card disease data set. After preliminary screening of 20 acting genes, combined with PPI network, GO, and K EGG enrichment analysis, 10 hub genes related to immune inflammation were identified, namely CXCL8, EGF, PTGS2, FOS, PECAM1, IL17A, TGFA, MMP1, CXCL5, and BCL2L1. Screening showed that Jak-STAT signaling pathway, Complement and coagulation cascades, IL-17 signaling pathway, Toll-like receptor signaling pathway, Pathways in cancer, EGFR tyrosine kinase inhibitor resistance, FoxO signaling pathway, mTOR signaling pathway, etc, are closely related to the occurrence of CP.

**Figure 7.**
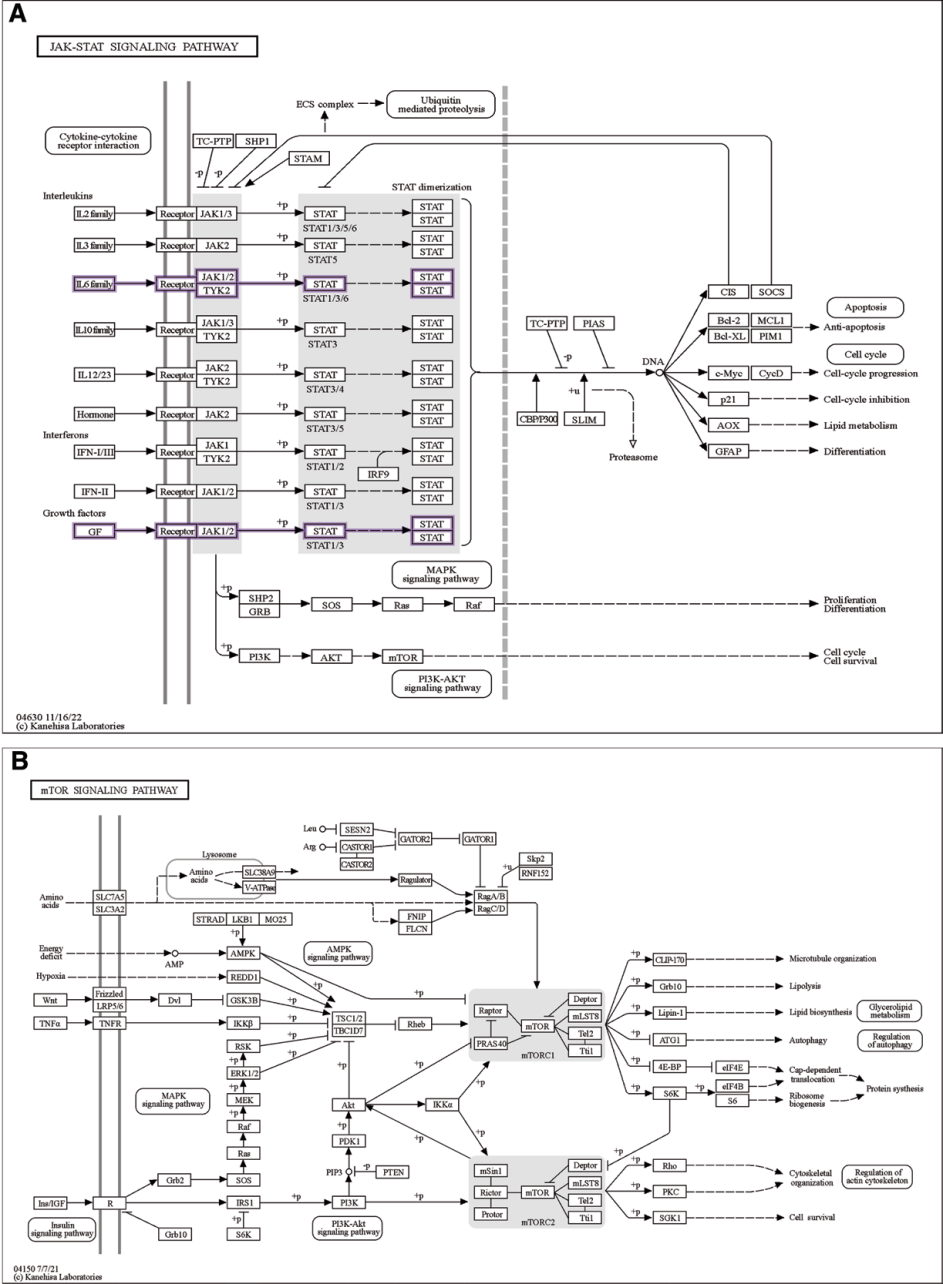
(A) Expression of Jak-STAT signaling pathway and related targets, (B) expression of mTOR signaling pathway and related targets.

Sustained and excessive cytokine production is a hallmark of inflammation and drives the pathogenesis and progression of chronic inflammatory diseases. Therefore, cytokine analysis can reveal the status of inflammation and immune system dysregulation, providing therapeutic targets for the treatment of cytokine-mediated diseases. In one study, higher levels of cerebral circulating cytokines, including IL-1β, IL-6, TNF, and CXCL8, were found to be associated with abnormal nervous system development. Additionally, another study found that inflammatory mediators drive neuroinflammation in autism spectrum disorder and CP. These inflammatory factors mainly include TNF-α, IFN-γ, GM-CSF, IL-4, IL-6, IL-17A, and IL-10. However, investigating potential associations between inflammatory molecules and neurodevelopment in children with CP requires further original research to elucidate the impact of prenatal and perinatal inflammation on neurological outcomes.^[30]^ PTGS2 is considered a target for relieving pain and treating inflammation. In the neuroinflammatory response, the expression of PTGS2 in astrocytes and microglia is significantly increased. PTGS2, as a neurotoxic mediator, may be responsible for neurodegeneration, psychiatric diseases, and pathological factors of epilepsy.^[31,32]^ Many studies have found that constitutive activation of STAT family members is directly related to tumor angiogenesis and other processes in cancer progression. In particular, STAT3 is closely related to the induction of pro-angiogenic factors in response to hypoxia and pro-inflammatory cytokines, and STAT5 induces the expression and secretion of pro-angiogenic factors.^[33]^ Jak-STAT signaling pathway is one of the main pathways of cytokine signal transduction. Related studies have found that the selective JAK2 inhibitor AG490 can significantly inhibit the phosphorylation of JAK2 and its downstream molecules STAT1 and STAT3. AG490 can significantly inhibit the expression of MCP-1 and ICAM-1 proteins in the kidney, and at the same time reduce the accumulation of macrophages in the kidney. Administration of AG490 immediately after ischemia can also significantly improve renal injury.^[34]^ Based on these studies and the results of this experiment, we predict that the Jak-STAT signaling pathway mediated by the STAT family plays an important role in the process of CP, and regulating related targets and pathways will help improve the occurrence of CP (Fig. 7A).

Various regulatory factors such as oxygen and nutrition are involved in fetal development, and mammalian target of rapamycin (mTOR) signaling is one of the main regulatory factors. mTOR signaling is involved in the regulation of various processes and plays an important role in protein translation, autophagy, cytoskeleton regulation, and gene transcription (Fig. 7B). In fetal growth restriction, placental agenesis can lead to reduced mTOR activity in the placenta and decidua, resulting in reduced placental blood flow, which may be one of the factors leading to CP.^[35]^

## 5. Conclusion

In summary, this study analyzed the differentially expressed gene data of children with CP through bioinformatics. The enrichment analysis of differential genes found that biological processes such as immune response and signaling pathways such as Jak-STAT signaling pathway and mTOR may be involved in CP. The pathogenesis of CP; by constructing a differentially expressed gene PPI network, we can obtain the core genes—CXCL8, EGF, PTGS2, FOS, PECAM1, IL17A, TGFA, MMP1, CXCL5, and BCL2L1—that may be involved in the pathogenesis of CP. The above targets and signaling pathways may have an important influence and role in the occurrence and development of CP and are worthy of in-depth exploration in the next step of research.

## Author contributions

**Writing—original draft:** Bo Chen.

**Formal analysis:** Ling Wang.

**Methodology:** Ling Wang.

**Visualization:** Dongke Xie.

**Conceptualization:** Yuanhui Wang.

## Supplementary Material
















